# The Composition and Predictive Function of the Fecal Microbiota Differ Between Young and Adult Donkeys

**DOI:** 10.3389/fmicb.2020.596394

**Published:** 2020-12-03

**Authors:** Jingya Xing, Guiqin Liu, Xinzhuang Zhang, Dongyi Bai, Jie Yu, Lanjie Li, Xisheng Wang, Shaofeng Su, Yiping Zhao, Gerelchimeg Bou, Manglai Dugarjaviin

**Affiliations:** ^1^Inner Mongolia Key Laboratory of Equine Genetics, Breeding and Reproduction, College of Animal Science, Equine Research Center, Inner Mongolia Agricultural University, Hohhot, China; ^2^College of Agronomy, Shandong Engineering Technology Research Center for Efficient Breeding and Ecological Feeding of Black Donkey, Shandong Donkey Industry Technology Collaborative Innovation Center, Liaocheng University, Liaocheng, China; ^3^National Engineering Research Center for Gelatin-based Traditional Chinese Medicine, Dong-E-E-Jiao Co., Ltd., Liaocheng, China

**Keywords:** donkey, age, gut microbiota, colonization, functional prediction

## Abstract

The community of microorganisms inhabiting the gastrointestinal tract of monogastric herbivores played critical roles in the absorption of nutrients and keeping the host healthy. However, its establishment at different age groups has not been quantitatively and functionally examined. The knowledge of microbial colonization and its function in the intestinal tract of different-age donkeys is still limited. By applying the V3–V4 region of the bacterial 16S rRNA gene and functional prediction on fecal samples from different-age donkeys, we characterized the gut microbiota during the different age groups. In contrast to the adult donkeys, the gut microbiota diversity and richness of the young donkeys showed significantly less resemblance. The microbial data showed that diversity and richness increased with age, but a highly individual variation of microbial composition was observed at month 1. Principal coordinate analysis (PCoA) revealed a significant difference across five time points in the feces. The abundance of *Bacteroides*, *Lactobacillus*, and *Odoribacter* tended to decrease, while the proportion of *Streptococcus* was significantly increased with age. For functional prediction, the relative abundance of pathways had a significant difference in the feces across different age groups, for example, Terpenoids and Polyketides and Folding, Sorting, and Degradation (*P* < 0.05 or *P* < 0.01). The analysis of beta diversity (PCoA and LEfSe) and microbial functions predicted with PICRUSt (NSTIs) clearly divided the donkeys into foals (≤3 months old) and adults (≥7 months old). Microbial community composition and structure had distinctive features at each age group, in accordance with functional stability of the microbiota. Our findings established a framework for understanding the composition and function of the fecal microbiota to differ between young and adult donkeys.

## Introduction

Monogastric herbivores are an important component of the agricultural sector, due to the meat, milk, and skin they produce for human use; however, these animals must consume increasing feed resources to meet the demands of the growing human population (Eisler et al., 2014). Consequently, improvement of feed efficiency is critical to the development of a sustainable monogastric herbivore. In particular, the major end products of the gut microbiota catabolism heavily depend on dietary fiber, i.e., the short-chain fatty acids acetate, propionate, and butyrate; these are a key energy source for the monogastric herbivores (Brokner et al., 2016). Indeed, the gut microbial systems, which aid in the digestion of otherwise indigestible nutrients, are the most complex biological system in animals and have been considered as an extra digestive organ (Eisler et al., 2014). A growing body of evidence demonstrates that the relatively stable function of the gut microbiota supplies the host with increased adaptability to the environment (Gao et al., 2020) and plays fundamental roles in intestinal physiological development and nutrient metabolism (Bäckhed et al., 2015; Wu et al., 2016). Therefore, understanding the gut microbiota of donkeys at different age in-depth not only can increase animal health, feed utilization efficiency, and production, but also may provide guidelines to early weaning technique.

Donkeys and horses (genus *Equus*) are typical monogastric herbivores. Almost all herbivores use crude fiber as a fermentation substrate in the intestine, which is the main activity location of microbiota (Bergman, 1990). Many diseases in the horse are related to the destruction of the intestinal microbiota, the most obvious being colitis (Harlow et al., 2013), laminitis (Moreau et al., 2014), and transient diarrhea (Kuhl et al., 2011) in young foals. At present, the gut microbiota of the newborn equine is already complex; for example, Satokari et al. (2008) isolated *Streptococcus* and *Enterococcus* from the umbilical cord and meconium of newborn foals. It has been shown that the delivery mode and breast-feeding strongly affect the development and function of the intestinal microbiota in humans (Bäckhed et al., 2015). The initial microbial colonization alters the intestinal environment to benefit the growth of specific anaerobes, which plays a dominant role in later growth and development (Favier et al., 2002). The gut microbiota thus clearly plays an important role for equine health and forage utilization. At present, most studies of equine intestinal microbiota focus on feces (Liu et al., 2014), different regions of the digestive tract (Schoster et al., 2017), and the characteristics of the microbiota in healthy and diarrheic animals (Costa et al., 2012). From an ecological point of view, early colonization of the gut microbiota represents the *de novo* assembly of a microbial community (Costello et al., 2012) and is influenced by animal species, age, and diet (Zoetendal et al., 2001; Torok et al., 2008; Carmody et al., 2015).

Donkeys are an important species as its skin could be used to produce Ejiao, a famous Chinese medicine, and are also considered as highly valued livestock due to their high-quality meat and skin. Now, the Chinese livestock industry includes approximately 2,500,000 donkeys. In previous studies, we have examined the different digestive tract microbiota of donkeys (Liu et al., 2019). However, limited information is known about the development of the bacterial community composition and function in the feces of donkeys at different ages. Therefore, in this study, we aimed to determine how microbial diversity and function changes in donkeys’ feces with age. The results of this study may shed new light on the microbiota–functional interactions at different ages.

## Materials and Methods

### Animals, Management, and Sample Collection

All procedures involving animals were approved and authorized by the Animal Welfare Committee of Liaocheng University. This study included 25 healthy DeZhou donkeys: they were divided into five groups (F1, F3, F7, F12, and F24) according to their age, and five heads in each group. Donkeys were kept outdoors at the Dong-E-E-Jiao Co., Ltd., national black donkey-breeding center (Donge county, Shandong Province) (June 25, 2019, temperature 23–34°C) and suckled until weaning (month 7). All donkeys had no gastrointestinal diseases or any antimicrobial exposure during the previous 3 months. They were fed a standard concentrate diet comprising 1.3% body weight twice daily (at 8:00 a.m. and 4:00 p.m.), and soybean straw (ratio of 60:40) had a free feeding (Burden and Bell, 2019). Meanwhile, they had free access to fresh water. Unweaned donkeys and their mother donkeys havea free access to soybean straw, while only unweaned donkeys could intake the concentration in their stables. Foals were weaned when they were at month 7 by physical separation from their mother.

The farm technician would collect fecal samples from donkeys after feeding 1 h at months 1, 3, 7, 12, and 24 after birth, picking feces out of the rectum with sterile gloves. Samples were immediately sealed in 50-ml aseptic cryopreservation tubes and put in liquid nitrogen and then stored at −80°C until DNA extraction.

## Bacterial DNA Extraction, Purification, and High-Throughput Sequencing

We extracted total genomic DNA from each fecal sample animal using a QIAam stool Mini Kit (Qiagen, Valencia, CA, United States), according to the manufacturer’s instructions. The purity and integrity of the DNA were checked using gel electrophoresis.

The bacterial primer pair 341F (5′-CCTAYGGGRBGC ASCAG-3′) and 806R (5′-GGACTACNNGGG TATCTAAT-3′) (Sonnenburg et al., 2010) were used to amplify the V3–V4 regions of the bacterial 16S rRNA gene (Liu et al., 2019) using Phusion High-Fidelity PCR Master Mix (NEB, New England Biolabs). Each primer contained the appropriate Illumina adapter sequence and 8-bp identifier indices. The resulting amplicons were purified using QIA quick Gel Extraction Kits (Qiagen, Valencia, CA, United States), and pooled in equimolar concentrations. Then, the purified amplicons were quantified using a fluorescence spectrophotometer (Thermo Scientific, MA, United States). Constructed libraries were subjected to Qubit quantitation and library testing, and then sequenced on an Ion S5TMXL system (Thermo Fisher Scientific, Franklin, MA, United States).

### Bioinformatics Analysis

Raw sequencing data typically contains a certain proportion of low-quality sequences. To ensure the accuracy and reliability of follow-up analyses, the original data (raw reads) must be quality controlled and filtered to obtain effective data (clean reads) (Haas et al., 2011). We removed low-quality reads using Cutadapt (v1.9.1) and barcode and primer sequences using Chromas. Sequences were spliced together using DNASTAR and removed chimeric sequences using Bellerophon (Huber et al., 2004).

The cleaned sequences were analyzed using QIIME (v1.7.0) (Caporaso et al., 2010) and clustered into operational taxonomic units (OTUs) based on 97% identity using UPARSE (Edgar, 2013). The representative sequences of each OTUs were aligned to the Greengenes database (DeSantis et al., 2006) using PyNast (Gregory et al., 2009). After that, the taxonomic information was annotated against the SILVA database using the RDP Classifier with a 0.80 confidence threshold (Wang et al., 2007; Christian et al., 2012). OTUs abundance information was normalized using a standard of sequence number corresponding to the sample with the least sequences.

To identify shared OTUs among groups, we constructed a Venn diagram using the Draw Venn Diagram online tool (Heberle et al., 2015). Alpha diversity indexes (i.e., Good’s coverage, Chao1, Ace, observed species, and Shannon) using QIIME (v 1.7.0) (Gregory et al., 2009) were used to compare the bacterial richness and diversity among groups. We identified statistically significant differences in alpha diversity, and microbial composition among donkeys of different ages was evaluated by one-way AVOVA. Beta diversity (i.e., differences in species complexity among samples) was determined based on principal coordinate analysis (PCoA) using weighted_unifrac and linear discriminant analysis (LDA) effect size (LEfSe); these analyses were performed using Microbiome Analyst, a comprehensive web server for comparative metagenomics (Achal et al., 2017). Community membership and structure were compared and visualized between pairs of samples using the PCoA. LEfSe was performed to identify the bacterial taxa that differed between pairs of groups (Segata et al., 2011). Taxa with an LDA score > 4 were considered important biomarkers of each group. We considered *P* value < 0.05 to indicate significant differences among groups.

### Predicted Function of the Gut Microbiome

To reveal the potential metabolic capabilities of the bacterial communities, we used PICRUSt (phylogenetic investigation of communities by reconstruction of unobserved states) to predict the function of gut microbiota based on the OTU table (Langille et al., 2013). Before PICRUSt analysis, we used the closed-reference OTU-picking protocol in QIIME against Greengenes to construct PICRUSt-compatible OTUs. Then, PICRUSt was used to predict functional pathways based on Kyoto Encyclopedia of Genes and Genomes (KEGG) annotations (Breiman, 2001; Segata et al., 2011) at levels 1 and 2 (Langille et al., 2013). We used the nearest sequenced taxon index (NSTI) to evaluate the accuracy of the predictions of the metagenomes. This algorithm used a phylogenetic tree of 16S rRNA gene sequences to associate OTUs with gene content. The relationships between functional capacities and predicted relative gene abundances were analyzed using PCoA and heatmaps. LSD-t was used to compare the relative abundance changes between different groups.

## Results

### Sequence Data and Alpha Diversity

**FIGURE 1:**
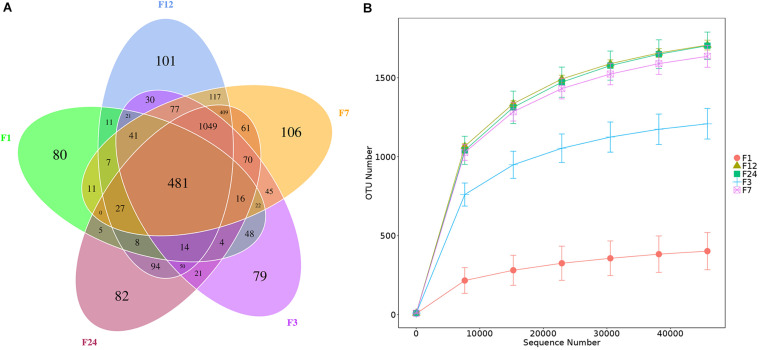
Numbers of bacterial operational taxon units (OTUs) in the feces of donkeys at different ages. Venn diagram, where areas of overlap indicate the numbers of OTUs shared among the overlapping groups **(A)**. Rarefaction curve showing the number of OTUs recovered at different sequencing depths **(B)**. F1: at month 1; F3: at month 3; F7: at month 7; F12: at month 12; F24: at month 24.

We obtained 1,655,064 16S rRNA gene sequences across all fecal samples. The sequences were clustered into 3,235 OTUs at 97% sequence identity: 26 phyla, 33 classes, 62 orders, 105 families, and 209 genera. Core OTUs are common OTUs in each sample. We identified 481 core OTUs in all samples and 448 OTUs unique to one age group: 80 OTUs unique to group F1, 79 OTUs unique to group F3, 106 OTUs unique to group F7, 101 OTUs unique to group F12, and 82 OTUs unique to group F24 (Figure 1A). The rarefaction curves across all samples tended to plateau at a sequencing depth of 45,802, indicating that this sequencing depth captured most of the microorganisms presented in the samples (Figure 1B). However, not only fewer OTUs consisted in the younger donkeys (groups F1 and F3) (*P* < 0.01), but lower sequencing depths were also required to cover the fecal microbiota at these ages (Figure 1B).

There were no significant differences in the numbers of effective sequences obtained across the 25 fecal samples (*P* > 0.05; Table 1). At a sequencing depth of 45,802, the Good’s coverage indexes ranged from 0.994 to 0.998, indicating that more than 99% of all bacterial taxa were captured across all samples. The species richness indexes (i.e., observed species, Chao1, and ACE) and the species diversity index (Shannon) for the older groups (F7, F12, and F24) were significantly higher than those in the F1 and F3 groups (*P* < 0.01). There were no significant differences in any indexes among older groups (*P* > 0.05). In addition, the Shannon index of F1 group was the lowest (4.43), indicating that the fecal microbial diversity of the F1 group was low and the microbiota was relatively simple.

**TABLE 1:** Sequencing statistics for the fecal microbiota of donkeys at different ages, including numbers of OTUs (97% similarity) and diversity indexes.

Items	Groups					*P* value
	F1	F3	F7	F12	F24	
Clean data (mean ± SE)	76,801.67 ± 5,787.39	81,435.00 ± 3,250.15	79,922.00 ± 14,60.17	80,129.00 ± 38.19	74,624.00 ± 7,456.86	<0.001
OTUs per sample (mean ± SE)	475.30 ± 162.70^C^	1,340.00 ± 122.3^B^	1,789.0 ± 86.4^A^	1,785.40 ± 47.83^A^	1,823.60 ± 130.2^A^	<0.0001
Good’s coverage (mean ± SE)	0.998 ± 0.000^A^	0.996 ± 0.000^B^	0.995 ± 0.000^C^	0.995 ± 0.001^C^	0.994 ± 0.001^C^	<0.0001
Chao1 (mean ± SE)	470.35 ± 156.47^C^	1,178.18 ± 341.12^B^	1,777.48 ± 88.17^A^	1,840.29 ± 67.92^A^	1,867.06 ± 112.60^A^	<0.0001
Ace index (mean ± SE)	495.35 ± 147.05^C^	1,199.44 ± 330.82*B*^B^	1,785.64 ± 80.57^A^	1,861.16 ± 67.71^A^	1,888.36 ± 515.55^A^	<0.0001
Observed species (mean ± SE)	401.00 ± 14.21^C^	1,078.60 ± 33.37^B^	1,639.40 ± 75.94^A^	1,703.60 ± 41.60^A^	1,689.60 ± 94.56^A^	<0.0001
Shannon (mean ± SE)	4.43 ± 0.88^B^	6.51 ± 2.53^AB^	8.55 ± 0.26^A^	8.31 ± 0.25^A^	7.89 ± 0.77^A^	<0.0001

*In the same row, values with different small letter superscripts mean significantly different (*P* < 0.05), those with different capital letter superscripts mean significantly different (*P* < 0.01), while those with the same or letter superscripts mean not significantly different (*P* > 0.05). F1, at month 1; F2, at month 3; F7, at month 7; F12, at month 12; F24, at month 24. SE, standard error. OTUs, operational taxon units.*

### Taxonomic Composition of Feces at Different Ages

**FIGURE 2:**
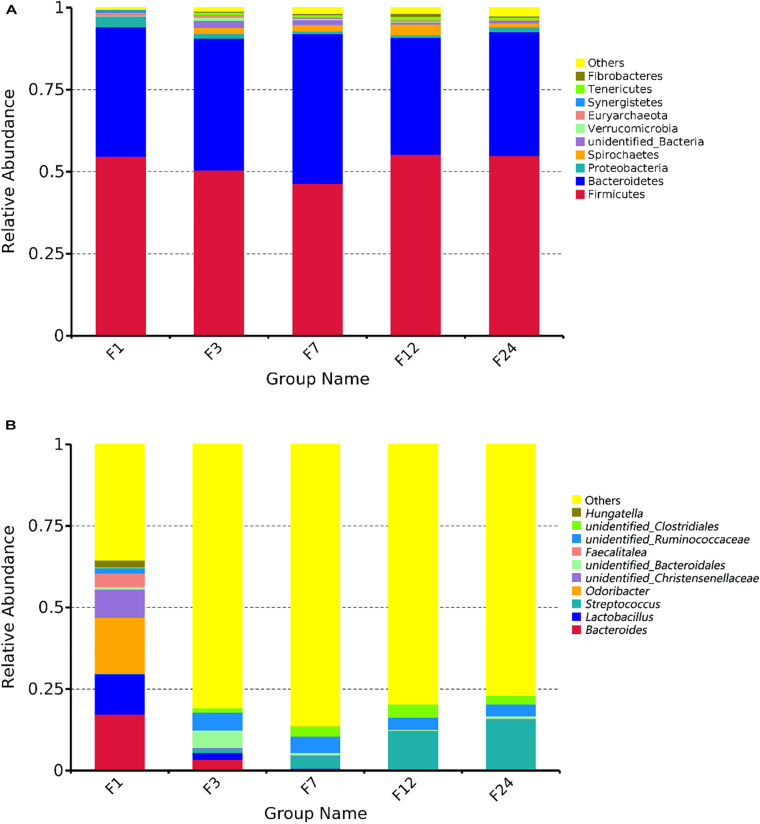
Microbial community composition in the feces of donkeys at different ages. Taxonomic composition at the phylum **(A)** and genus levels **(B)**.

We identified the composition of the top 10 bacterial phyla in the fecal samples (Figure 2A). The phyla *Firmicutes* and *Bacteroidetes* were the most abundant, together accounting for about 90% of the OTUs identified across all groups. *Firmicutes* and *Bacteroidetes* comprised 54.79 and 39.38%, respectively, of the bacteria in group F1; 50.54 and 40.13%, respectively, of the bacteria in group F3; 46.45 and 45.64%, respectively, of the bacteria in group F7; 55.25 and 35.78%, respectively, of the bacteria in group F12; and 54.88 and 37.86%, respectively, of the bacteria in group F24 (Figure 2A). Although genus with relative abundances less than 1% were most common in all age groups, group F1 had fewer of these genera (35.50%), and much higher relative abundances of *Bacteroides* (17.34%), *Odoribacter* (17.20%), *Lactobacillus* (12.31%), and unidentified *Christensenellaceae* (8.59%) (Figure 2B). In groups F3–F24, genera with relative abundances less than 1% accounted for 80.79, 86.32, 79.51, and 76.90% of all bacteria, respectively, with the remaining bacteria primarily *Streptococcus*, unidentified *Ruminococcaceae*, and unidentified *Clostridiales*. Interestingly, group F3 retained some *Bacteroides* (3.45%) and *Lactobacillus* (2.19%), which were not present in the older samples. Also, the relative abundance of *Streptococcus* increased with age. Then, we compared the relative abundance of these taxa associated with the different ages (Supplementary Figure 1). The proportion of *Bacteroides*, *Lactobacillus*, and *Odoribacter* reduced linearly from groups F1 to 24 (*P* < 0.05 or *P* < 0.01, Supplementary Figure 1B). The abundance of *Streptococcus* was significantly higher at groups F12 and F24 than at groups F1 and F3 (*P* < 0.01), and there was no significant difference with group F7 (*P* > 0.05).

**FIGURE 3:**
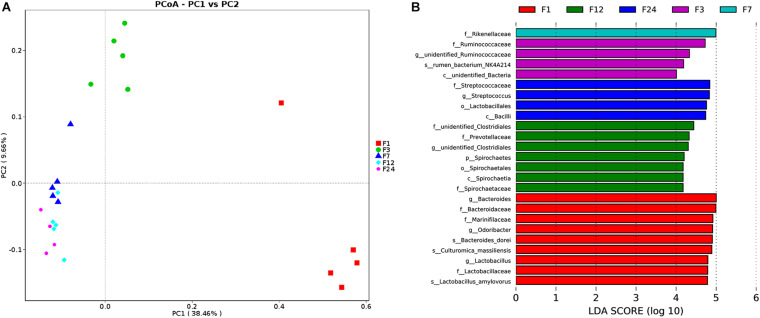
Alterations in fecal microbiota of donkeys at different ages. Principal coordinate analysis (PCoA) showing phylum-level differences **(A)**. Histogram showing taxa with statistically significantly linear discriminant analysis (LDA) values of donkeys at different ages **(B)**. f, family; g, genus; s, species; o, order.

PCoA revealed that the microbial communities from feces across different ages are varied (Figure 3A). The unweighted unifrac distance analysis showed that the community composition of the feces at groups 1 and 3 was significantly separated from other groups (at groups 7, 12, and 24) (AMOVA < 0.01); PC1 explained 38.46% of the variation among groups.

We employed a LEfSe analysis to identify the taxa that most strongly differentiated the age groups. More differential bacteria were found through comparing to other groups. At group 1, there were several microbial populations that were enriched; these included *Bacteroidaceae*, *Marinifilaceae*, *Odoribacter*, and *Lactobacillaceae*. Only one family was found at groups 3 (*Ruminococcaceae*) and 7 (*Rikenellaceae*), respectively. In comparing the different bacteria of fecal samples at groups 12 and 24, there were proportional differences between *Prevotellaceae* and *Spirochetes* at group 12, and *Streptococcaceae*, *Lactobacillaceae*, and *Bacilli* at group 24 (Figure 3B).

### Functional Predictions of the Rectal Microbiota Using PICRUSt

**FIGURE 4:**
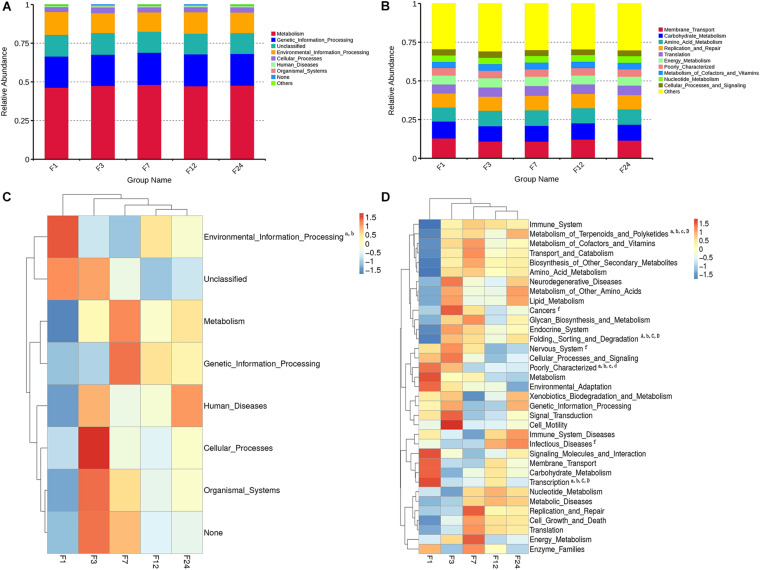
Relative enrichment of KEGG Level 1 **(A)** and Level 2 **(B)** pathways in fecal microbiota of donkeys at different ages. Corresponding heatmaps for KEGG Level 1 pathways **(C)** and KEGG Level 2 pathways **(D)**. Differences in relative abundance of each functional gene were examined using one-way ANOVA, and letters (a, b, c, d, e, f, and g) indicate significant differences between groups 1 and 3; between groups 1 and 7; between groups 1 and 12; between groups 1 and 24; between groups 3 and 7; between groups 3 and 12; and between groups 3 and 24, respectively. Lowercase letters correspond to significant differences (*P* < 0.05); capital letters correspond to extremely significant differences (*P* < 0.01).

We predicted the function of the fecal microbiota of donkeys at different ages using PICRUSt, and we used the NSTIs to evaluate the accuracy of these predictions. Metabolism was the most overrepresented level 1 pathway across all groups, while membrane transport, carbohydrate metabolism, amino acid metabolism, and replication and repair were among the most overrepresented level 2 pathways (Figures 4A,B). However, of the level 1 pathways, environmental information processing was significantly more common in group F1 as compared to groups F3 and F7 (*P* < 0.05) (Figure 4C).

**FIGURE 5:**
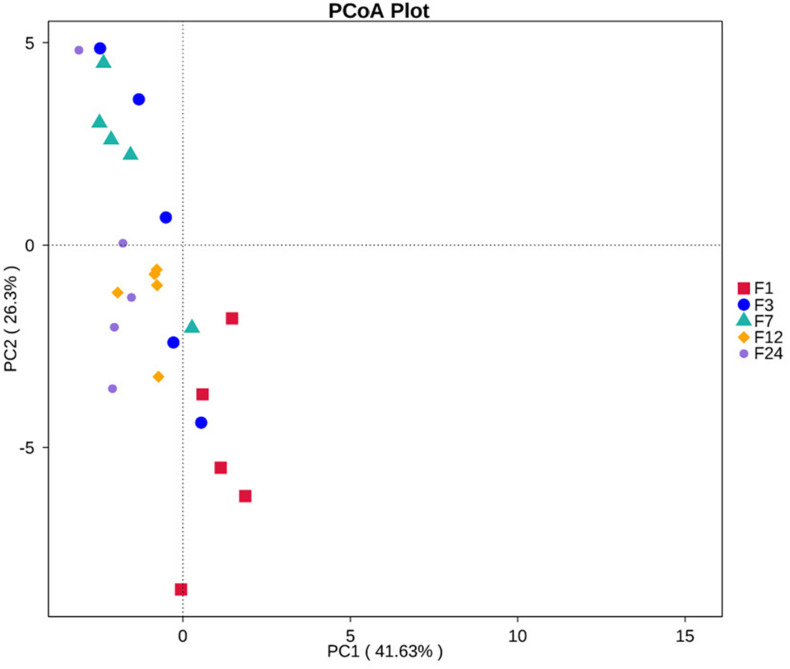
PCoA of KEGG Level 2 pathways in fecal microbiota of donkeys at different ages.

Of the 35 level 2 KEGG pathways, the relative abundance of Membrane Transport, Carbohydrate Metabolism, and Amino Acid Metabolism were at the highest level during different ages. The result shows that metabolism of Terpenoids and Polyketides and Folding, Sorting, and Degradation were significantly gradually enriched from group F1 to group F24 (*P* < 0.05 or *P* < 0.01), while Poorly Characterized and Transcription pathways were significantly gradually reduced from group F1 to group F24 (*P* < 0.05; Figure 4D). Cancers, Nervous system, and Infectious diseases pathways were significantly enriched in group F3 as compared to group F12 (*P* < 0.05). The 35 level 2 KEGG pathways among F7, F12, and F24 had no significant difference (*P* > 0.05), which indicated that the prediction function of microorganisms in rectal contents at 7 months is similar to that of adult donkeys, and 7 months of age is a transitional stage. PCoA revealed that the functions of the fecal microbiota from different ages were clustered, with the first two components explaining a total of 67.93% of the variation. The results suggested that the functional KOs at group F1 were distinctly separated from other groups, explaining 41.63% variation (Figure 5).

## Discussion

In the present study, we described the early colonization process of the donkey gut microbial ecosystems, on the basis of different age. When searching for bacterial OTUs shared among all groups, we were successful in detecting 481 OTUs shared, and each group has its own unique OTUs. The existence of OTUs specifically shared among all groups suggests that each of them contained similar bacterial community, and there was also specificity in terms of microbial DNA components. We also found that microbial diversity in the feces from the youngest donkeys (month 1) was highly variable as compared to those of older donkeys. The species richness and Shannon indexes were higher at 7, 12, and 24 months than at 1 or 3 months, indicating an increase in richness with developmental age. This result was consistent with previous investigations of the feces, ilea, or rumens of foals (Costa et al., 2016), goats (Jiao et al., 2016), deer (Li et al., 2018), and cattle (Uyeno et al., 2010; Georgios et al., 2013) at different ages. Previous studies have indicated that microbial diversity in the gut may be influenced by many factors, including the characteristics of the mother’s milk (in the case of suckling animals) (Mueller et al., 2015), the forage eaten (Harlow et al., 2016), sex (Mshelia et al., 2018), age (Stephens et al., 2016), and the microbiota of the surrounding environment (Chen et al., 2018; Zhou et al., 2019). It has been shown that the bacterial communities in human breast milk are similar to the infant gut microbiota within a few days of birth (Williams et al., 2019), and infants can receive 27.7% of the bacteria from breast milk during the first 30 days of life after receiving 75% of total daily milk intake as breast milk (Collado et al., 2007). However, the components of mother’s milk play a more important role in the early colonization of the intestine with the start of mother’s milk (Quercia et al., 2019), including protein, lactose, milk fat, and other nutrients. Newborn donkeys only have a single stomach and can be physiologically and functionally considered non-ruminants, so the mother’s milk can pass directly to the small intestine. Perhaps this is one of the reasons why microorganisms in the feces of young animals differ from those of adults. The impact of mother’s milk on microbial composition requires further investigation.

The phyla *Firmicutes* and *Bacteroidetes* are dominating in the feces over the development of these donkeys. This is consistent with a previous study of the feces of DeZhou donkeys (Liu et al., 2014) and disagrees with the results from the feces of 2-week-old healthy foals (Schoster et al., 2017). This incongruence suggests that the relative abundances of certain bacterial phyla may differ among the feces of animals in the same genus. The *Enterococcus* and *Enterobacteriaceae* were found in meconium from birth to the third day of life, indicating that the foal gut microbiota can acquire microorganisms typical of the milk community (Quercia et al., 2019). These results imply that the mother’s milk is an important factor to affect intestine microbial composition. This experiment showed that *Bacteroides*, *Lactobacillus*, and *Odoribacter* are the dominant bacteria in young donkeys (months 1 and 3), and these bacterial genera trend to decrease with age, while *Streptococcu*s is the most abundant bacterial genus in adult donkeys (months 7, 12, and 24). The bacterial composition in feces of different ages was consistent with LEfSe analysis results. The different composition of the genus may be influenced by the mother’s milk while reinforcing the idea that the early microbiota in the feces is related to the milk and the environment. As crude fiber intake increased with age, *Streptococcus* became the dominant bacterial genus, presumably to break down large volumes of cellulose. However, these hypotheses need to be documented in a future study.

According to our findings, the donkeys’ gut microbial ecosystems describe a specific developmental trajectory from infancy to adulthood, progressively approaching the configuration typical of the adult gut, especially starting from 1 month old to 7 month old. The temporal changes in the gut microbiota of donkeys characterized here indicated that the intestinal flora develops as the donkey ages. In *Marinifilaceae* and *Odoribacter*, which were found to dominate at month 1, the bacteria characteristics of the feces were suddenly lost with the start of mother’s milk intake. The high abundance of subsequently uncommon bacterial genera at month 1 might be due to environmental exposure or to the wide range of bacteria to which the foal is exposed through the mother. In infants, the first change of fecal bacterial composition occurs around 5 days of age; it will transform to an adult-like pattern and usually after eating solid food (Bäckhed et al., 2015). In contrast to human, donkeys start to intake small amounts of concentrates already around 10 days of age (Yatsunenko et al., 2012). To our knowledge, the donkeys intake more and more concentrate and roughage with age, so the gut microorganisms of donkeys tend to be more stable with age and more resistant to diseases. The results showed that these taxa do not represent the true colonizers until by month 7, but only transient organisms. The supposition is supported by the quick reduction in the relative abundances of many of the initial microbial genera, as the true colonizers begin to take hold. Our results indicated that month 7 represents a critical period in the intestinal microbiota development, because, by this point, the structure of the intestinal microbiota was similar to that of an adult donkey. From month 7, the donkey gut microbial ecosystems begin to converge to the adult one. This process involves the loss of special microorganisms and the concomitant acquisition of fiber fermenters typical of adult core gut microbiota, such as *Prevotellaceae*, *Spirochetes*, *Streptococcaceae*, and *Lactobacillaces*. These data indicated that, by month 7, the donkey’s gut microbiota has begun to build up gradually to include the bacteria necessary for the digestion of the roughage found in the adult donkey diet, much like what had been shown in a study of weaning (day 60) and adult (mare) horse (De La Torre et al., 2019). Meanwhile, it is feasible to choose weaning at the age of 7 months in production practice, which not only can ensure the stability of intestinal microorganism but also can reduce the incidence of disease.

PICRUSt functional prediction analysis indicated that, in donkeys of all ages, the membrane transport, carbohydrate metabolism, and amino acid metabolism at level 2 were the most overrepresented. It has been discovered that these pathways exactly were the most active genes in the intestinal tract (Liu et al., 2019). We found that environmental information processing and Poorly Characterized and Transcription pathways were more enriched in the young donkeys at months 1 and 3, while metabolic pathways (i.e., amino acid, energy, enzyme families, and vitamins) were more enriched in the adult donkeys at months 12 and 24. The old donkeys require more energy to survive, so the function of metabolic pathways was enhanced. Also, Cancers and Nervous system pathways were enriched in group F3, and we speculate that the increased intake of forage caused a disturbance in the gut microbiome that stimulated the immunity system of donkeys and drove the inflammation. The differences of microbial function at different ages indicated that the varied pathways enriched in the young donkeys might help to adapt to the post-birth environment and that microbial functions are known to vary throughout the host’s life, due to the interactions between the host and the microbes (Newell and Douglas, 2014). This was consistent with our results; meanwhile, the 35 level 2 KEGG pathways among months 7, 12, and 24 had no significant difference, which suggested that donkeys’ age correlated with differences in the composition, diversity, and function of the fecal microbiota due to the adaptations of the microbiota to their rapidly changing environment. Here, the functional KOs at month 1 was distinctly separated from other groups, indicating that there was a significant difference at month 1 compared with other months and suggesting a shift toward a more adult-like intestinal environment associated with the increased functional capacity for metabolic pathways. Collectively, these results indicate that both the composition and the function of the gut microbiota evolve as the donkeys grow, in response to environment factor and resources feeding into the community. The limitation of the present study is that the metagenomic shotgun sequencing of the feces was not examined, which will provide more accurate and direct evidence to the function of the gut microbiota in future studies.

## Conclusion

In summary, we analyzed the fecal microbiota of donkeys at different ages using high-throughput 16S rRNA bacterial gene sequencing and PICRUSt. Our results showed that the microbial diversity in the feces increased as the donkey aged, indicating that age was a key factor in microbial succession. Microbial community composition and functional prediction in the feces of young donkeys (groups F1 and F3) differed significantly from those of older donkeys (groups F7, F12, and F24), implying that the component of mother’s milk influenced the composition of the initial gut microbiota. The results of the five time points revealed that the metabolic pathways changed with age and were more enriched in the adult donkeys at months 12 and 24. Meanwhile, month 7 represented a critical period in the intestinal microbiota development; from month 7, the donkeys’ gut microbial ecosystems began to converge to the adult one. These results increased our understanding of the age-specific variations in the fecal microbiota of donkeys, but additional studies are required to explore the relationships among microbial changes at different ages.

## Data Availability Statement

The datasets generated for this study can be found in NCBI SRA accession PRJNA673698, https://www.ncbi.nlm.nih.gov/bioproject/PRJNA673698/.

## Ethics Statement

The animal study was reviewed and approved by the Animal Welfare Committee of Liaocheng University.

## Author Contributions

JX and GL: laboratory work and writing of the manuscript. SS and DB: experimental development. JY and LL: providing donkeys and sample collection. XW, SS, and YZ: bioinformatic and statistical analyses. GB and MD: study design, interpretation of data, critical review, and final approval of the manuscript. All authors contributed to the article and approved the submitted version.

## Conflict of Interest

JY was employed by the company Dong-E-E-Jiao Co., Ltd. The remaining authors declare that the research was conducted in the absence of any commercial or financial relationships that could be construed as a potential conflict of interest. The authors declare that this study received samples from Dong-E-E-Jiao Co., Ltd. Dong-E-E-Jiao Co., Ltd was not involved in the study design, collection, analysis, interpretation of data, the writing of this article or the decision to submit it for publication.

## Supplementary Material

Supplementary Figure 1 Heatmaps of phylum **(A)** and genus **(B)** levels abundance. Differences in relative abundance of microbiota at genus level were examined using one-way ANOVA. Letters (a, b, c, d, e, and f) indicate significant differences between groups 1 and 3; between groups 1 and 7; between groups 1 and 12; between groups 1 and 24; between groups 3 and 12; between groups 3 and 24 respectively. Lowercase letters correspond to significant differences (*P* < 0.05); capital letters correspond to extremely significant differences (*P* < 0.01).
