# Supplementary figures and images for: The Composition and Predictive Function of the Fecal Microbiota Differ Between Young and Adult Donkeys
Source: Front Microbiol. 2020 Dec 3;11:596394. doi: 10.3389/fmicb.2020.596394 (PMC7744375; doi:10.3389/fmicb.2020.596394)

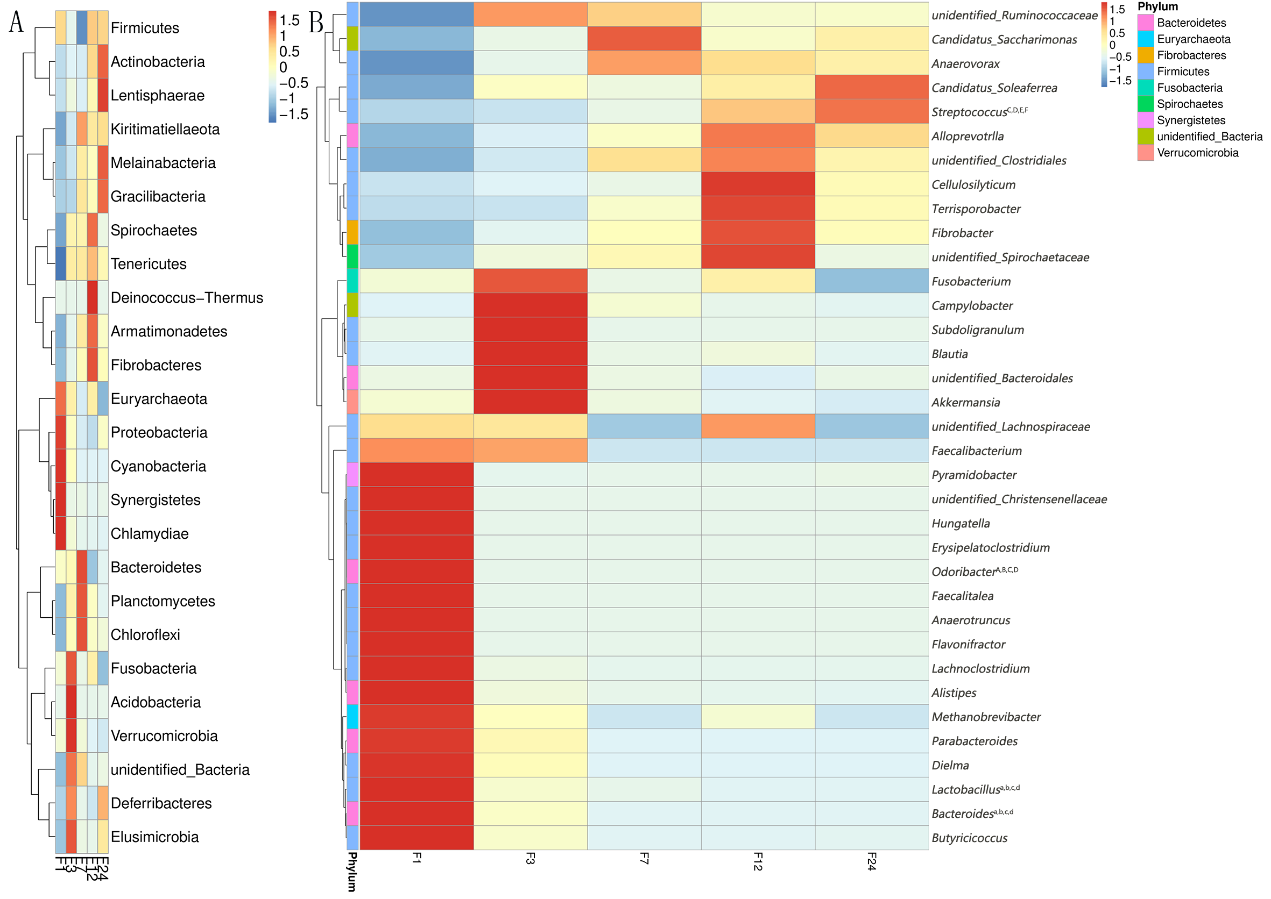

Supplement: Supplementary Figure 1 — Heatmaps of phylum (A) and genus (B) levels abundance. Differences in relative abundance of microbiota at genus level were examined using one-way ANOVA. Letters (a, b, c, d, e, and f) indicate significant differences between groups 1 and 3; between groups 1 and 7; between groups 1 and 12; between groups 1 and 24; between groups 3 and 12; between groups 3 and 24 respectively. Lowercase letters correspond to significant differences (P < 0.05); capital letters correspond to extremely significant differences (P < 0.01). [file Image_1.TIF]
